# Bridging the Research to Practice Gap: Feasibility of Implementing Social Rhythm Therapy for Bipolar Disorder in Specialist Mental Health Services

**DOI:** 10.1007/s10597-026-01613-w

**Published:** 2026-03-19

**Authors:** Maree L Inder, Marie T Crowe, Richard J Porter

**Affiliations:** University of Otago, Christchurch, New Zealand

**Keywords:** Social Rhythm therapy, Circadian rhythms, Implementation, Mental health services, Case management

## Abstract

Introduction. Despite research evidence supporting adjunctive psychological treatment for bipolar disorder, there is often a failure in translation to routine clinical care. This study aimed to determine the feasibility of implementing social rhythm therapy, a brief intervention with potential for adoption within Specialist Mental Health Services for individuals with bipolar disorder. Method. Case managers from two community mental health teams were trained to deliver social rhythm therapy to clients with bipolar disorder on their case load. Feasibility was based on qualitative and quantitative data addressing acceptability of training, ability to deliver therapy as part of a caseload, and adherence and fidelity for case managers, and acceptability of therapy including recruitment and retention for case managers and clients. Descriptive analyses were used for quantitative data and thematic analysis for qualitative data. Results. Ten case managers were trained with eight delivering the therapy. Of 37potential clients with bipolar disorder, 10 began therapy (27% uptake),with five completing (50% completion). Qualitative findings demonstrated acceptability of training with implementation challenges associated with engaging clients and balancing case management requirements with high workplace demands. Limitations included the small sample size and significant gaps in data, especially reflecting the clients’ perspectives. Conclusion. The feasibility of implementing of social rhythm therapy in Specialist Mental Health Services was impacted by low level of uptake and systemic barriers, primarily related to the requirements inherent in the case manager role. Effective implementation requires addressing the systemic issues and enhancing engaging individuals with bipolar disorder in psychological treatment approaches.

## Introduction

Large numbers of evidence-based psychosocial interventions for mental health care exist which can confer demonstrable effects on community-level outcomes (Daleiden et al., 2006). However, it is well established that many interventions found to be effective in research trials fail to translate into clinical practice (Girlanda et al., 2017). Clinical practice often lags behind evidence from clinical trials, with efforts to implement into everyday mental healthcare often unsuccessful (Kristensen et al., 2016). This has prompted research into reducing the “research-to-practice” gap and the growth of implementation science (Bauer et al., 2015; Thornicroft & Tansella, 2009).

Bipolar disorder (BD) is a serious, highly relapsing mood disorder with episodic periods of depression and hypomania or mania. The diagnostic criteria for depression includes depressed mood and or loss of pleasure in combination with other symptoms including sleep disturbance (insomnia or hypersomnia), reduced energy, appetite and weight changes, and suicidal thoughts for a minimum of two weeks. Mania involves the presence of elevated mood with concomitant symptoms of decreased need for sleep, grandiosity, increased goal-directed activity lasting for a period of a week or more. Hypomania has similar symptoms of lesser duration and level of impairment (American Psychiatric Association & American Psychiatric Association, 2013) BD is associated with a high degree of morbidity, disease burden, impairment of functioning and reduced quality of life (Vieta et al., 2018).

There is an increasing focus on the use of adjunctive psychological treatment for BD, such as Interpersonal and Social Rhythm therapy (IPSRT), given evidence by meta-analyses suggesting symptom improvement and reduced relapse rates of 40–50% (Oud et al., 2016; Scott et al., 2007), plus endorsement of their use in Mood Disorder Guidelines (Malhi et al., 2021; Yatham et al., 2018). Despite this, the availability of these therapies in clinical practice settings is limited (Barbato et al., 2016; Jaffé et al., 2024). IPSRT combines interpersonal therapy addressing interpersonal based stressful life events with a behavioural intervention stabilising social rhythms or routines (Frank, 2007; Novick et al., 2024). There is evidence supporting reduction in relapse rates (Frank et al., 2005), and improvement in symptoms and functioning (Crowe et al. 2020a, b; Inder et al. 2015; Steardo et al. 2020).

However, implementing IPSRT in clinical settings such as mental health outpatient clinics has identified challenges related to engaging clients, given the length and requirements of the therapy, transitioning from training to the delivery of IPSRT, fit with usual practice, and applying the model in the context of competing demands (Hamm et al., 2015; Stein et al., 2013).

A potential option that may be more suited to delivery in mental health services is Social Rhythm Therapy (SRT), a derivative of Interpersonal and Social Rhythm therapy (IPSRT) (Crowe et al. 2020a, b). There is emerging evidence that SRT may be effective without the interpersonal component and is supported by robust evidence of the importance of targeting circadian rhythms in BD (Gottlieb et al., 2019; Milhiet et al., 2011; Wirz-Justice, 2006). The social rhythm aspect has been identified as the most enduring and helpful aspect of IPSRT therapy by individuals with BD (Crowe & Inder, 2018; Hamilton & Inder, 2025). SRT also has a number of characteristics that suggest its applicability as intervention in clinical settings including short duration, behaviourally based, amenable to brief training, and flexible to individual patient needs (Crowe et al. 2020a, b). It has previously been used as a standalone therapy as an internet-based intervention in a primary health setting (which included supported clinical help) (Swartz et al., 2021) and a telephone delivered intervention in an outpatient clinic delivered by psychologists (Corruble et al., 2016).

Drawing on implementation principles (Greenhalgh et al., 2014; Lyon & Koerner, 2016), and based on the attributes of SRT suggesting its potential as a translatable intervention applicable to mental health services, the aim of this study was to determine the feasibility of implementing SRT for individuals with BD receiving outpatient care within Specialist Mental Health Services (SMHS).

## Method

### Process and Setting

The study was a collaboration between a publicly funded services SMHS catering for people with moderate to severe mental health conditions, and the research team. SMHS wanted to address the limited availability of psychological interventions, specifically a psychotherapy for BD delivered by case managers (CM) that could be implemented into routine clinical practice without changing the basic service structures. The study was undertaken in two multi-disciplinary Community Mental Health teams (CMHT). Usual treatment entails a psychiatrist providing medication management and a CM (psychiatric nurse, social worker or occupational therapist) largely responsible for all other aspects of treatment and care including monitoring mood state, encouraging medication adherence, and engaging in psychoeducational and supportive activities. Clinical psychologists provide additional therapy to patients with more complex needs as well as clinical support and guidance to the multidisciplinary team.

### Study Participants

CMs from two CMHT were trained in SRT with representatives from each discipline (nursing, social work, and occupational therapy) with half having experience of five or less years in mental health. The managers of all participating CM supported their involvement and the need for consideration of their caseload capacity.

All individuals with a diagnosis of BD on the caseload of participating CM and likely to remain with the service over the subsequent 12-weeks were invited by their CM to participate. The CM provided the Information Sheet and obtained consent.

#### Intervention

SRT is a behavioural intervention and focuses on the stabilization of social rhythms defined as the social relationships, social demands or tasks that serve to entrain biological rhythms (Ehlers et al., 1988). The intervention was manualised (Crowe, 2018) and covered the theoretical underpinnings of SRT, the structure and content of the sessions, and SRT-specific tools and resources. Twelve sessions of SRT were to be delivered over a flexible period.

### Training and Fidelity

The training involved eight hour online training through the IPSRT website (http://www.ipsrt.org), focusing specifically the six most relevant modules for SRT with the option of completion of the full 12 modules and test to gain an IPSRT certificate. A one-day in person practice-focused workshop. covered the content of the manual including the theoretical underpinnings and evidence for SRT, the focus and content of SRT for the beginning, middle and end of therapy, and involved the use of role plays and practice focused videos. Training and fortnightly supervision were provided by an experienced SRT researcher and a senior SMHS clinical psychologist. The supervision entailed ensuring adherence to SRT and addressing challenges in its delivery within the SMHS setting. There was a requirement for all cases to be presented by the CMs through all stages of the therapy process for each client. The clinical psychologist was also available to provide support and guidance to CM between supervision sessions. CMs completed a therapy tracking form recording the date, session number, and SRT focus for each session. CMs were required to tape therapy sessions from the beginning, middle, and end of therapy. The tapes were reviewed by the experienced SRT researcher with fidelity based on the alignment with the expected content for each phase of the therapy as set out in the SRT manual.

#### Outcome Measures

1. *Acceptability of training* - CM uptake of training and themes derived from semi-structured qualitative interviews with questions including the process and content of training delivery, gaps, and level of preparedness for delivery of SRT.
2. *Acceptability of therapy* - recruitment and retention of individuals with BD, and themes derived from semi-structured qualitative interviews on their experiences of receiving SRT covering their understanding of SRT, perceived value and impact, and reservations. CM also recorded any factors affecting retention of individuals in therapy.
3. *Ability to deliver SRT as part of caseload* **-** number of SRT sessions, therapy duration, and themes derived from semi-structured qualitative interviews of the CM on their ability to deliver SRT post training, barriers to delivery, suggested areas for change.
4. *Adherence and treatment fidelity* **–** attendance at fortnightly supervision, therapy tracking recording date and length of each SRT session as per SRT manual, and review of recorded sessions.

Individual qualitative interviews were undertaken by a clinical research nurse with CMs and clients who was not involved in the delivery of the intervention.

### Data Analysis

Descriptive analyses were undertaken for quantitative data including frequencies, percentages and ranges.

Qualitative interviews were transcribed and analysed using thematic analysis (Crowe et al., 2015) (Braun & Clarke, 2006). This involved finding patterns of meaning across the data through a series of steps: (1) in-depth reading of transcripts for meaning (2) identifying codes (3) grouping codes into categories of meaning (4) development of themes (5) interpretation of themes.

## Results

Ten CMs consented to participate with four registered nurses (RN), five social workers (SW) and one occupational therapist (OT), with half having less or equal to 5 years’ experience. All ten completed the training, with eight case managers progressing to deliver SRT. Of these eight, three case managers discontinued involvement with one going on maternity leave and two changing roles within SMHS.

## Acceptability of Training

Two themes were identified from qualitative interviews of CM’s experiences of the training, Preparedness to deliver SRT and Seeing potential of SRT.

*Preparedness to deliver SRT* captured how training supported the CM readiness to implement SRT. One of the key elements that was valued by CMs was how the training built on their prior knowledge and practice deepening their understanding of what they currently do and rationale for SRT.

*It coincides with a lot of things that we’ve sort of done but more in depth which is awesome because it gave more reason and purpose to why we need to be doing it.*

Understanding the circadian basis of SRT gave deeper meaning and legitimacy to routine strategies such as sleep hygiene.

*just the basic sleep hygiene and behaviour observations…I have always known that it’s something really important that we need to be doing*,* and it’s something that we talk about with our clients but having that more deeper about the circadian rhythms*,

The inclusion of practice elements such as videos, role plays and a structured manual was valued as it facilitated consolidation and guidance for the delivery of SRT as reflected by the CM.

*I think it’s really good [the training]. It’s that whole onion peeling back the layers consolidate learning isn’t it and getting that deeper knowledge of what we do and especially how we present it*,* and talking about it. It gives a really good break down of how we can do it.*

There were reservations on the online training element, however there was a recognition and appreciation of the benefits of this more flexible format.

*I wasn’t expecting it to be all on the computer*,* and I thought maybe it wouldn’t be that helpful*,* but I think it was really helpful being able to go at my own pace*,* to stop and start*,* to have breaks when I needed to*,* so I did really like that*.

The training achieved a level of preparedness reflected as being “confident enough” and a willingness to progress to implement SRT as described by this CM:

The best part would be just getting going and starting

### Seeing Potential of SRT

Seeing potential of SRT reflects the possible benefits of delivering SRT in their CM role

CM saw how their clients could benefit from the implementation of SRT and the strategies used in this therapeutic approach. This CM identified the social rhythm metric as a valuable tool which could help their clients to effectively focus on addressing routines:

*Getting the client to write down what their routines and their rhythms*,* I think that’s a really good intervention for them to also see some of those patterns as well that are there and have that discussion with them*.

Additionally, the focus on individualised goal setting was seen as positive and an empowering approach.

*Really encouraging the client to come up with sort of their own goals as well. I mean obviously that’s what we try and do in our work anyway but that being the focus because I think that’s quite empowering for people as well. To be able to [have] a bit of control in parts of their life*.

CMs also considered client related factors that may influence the suitability of SRT including such as their clinical state and their openness to engaging in a different treatment approach.

*I think it does depend on who you’re actually working with*,* someone who has quite a severe bipolar disorder or someone who is in the sort of lighter range and they’re sort of cognitive abilities as well and how entrenched they are in habits and whether they’re open to change.*

However, there were also concerns expressed about the competing demands of the CM role and how this will impact on effectively implementing SRT to their clients.

*It would just be that time commitment I think and if something comes up then what takes priority*,* you know as case manager you have to do the other stuff.*

Overall, there was an expressed value of CM delivering SRT as part of their role tempered with some caution related to the practicality of this.

*I think it would be really good in an ideal world. If all CMs could be doing it I think it would be really good.*

### Acceptability of Therapy

**Fig. 1:**
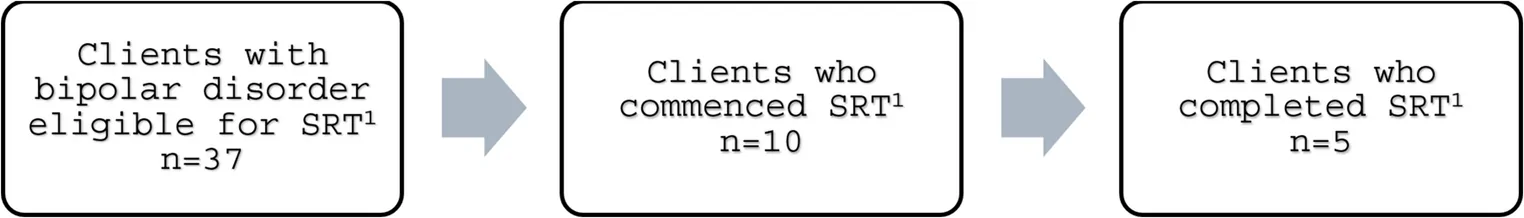
Participant flowchart for social rhythm therapy

The CMs identified 37 potential participants with a diagnosis of BD on their caseload with 10 clients progressing to commence SRT equating to an uptake rate of 27%. Factors influencing client uptake were varied with the main reason clients not interested/willing (*n* = 5), too unwell (*n* = 5), and due for discharge (*n* = 5). Five individuals completed SRT equating to a completion rate of 50%. (see Fig. 1).

The number of SRT sessions ranged from 10 to 13 with mean 11.2 (SD ± 1.3) and duration of therapy ranged from 23 to 63 weeks with a mean of 42 (SD ± 15.2) weeks. This equates to one SRT session per 3–4-week period.

The acceptability of therapy from the client perspective was unable to be assessed due to lack of data with only one client completing an interview.

## Ability to Deliver SRT as Part of a Caseload

Three themes were identified from qualitative interviews of CMs’ experiences of implementing SRT: Variable receptivity to SRT, Navigating dual roles, and Beyond medication.

*Variable receptivity to SRT* reflected the CMs experiences in engaging their clients to engage in SRT.

Receptivity to SRT was shaped by client contextual factors such as timing, level of wellness, and openness to alternative therapeutic approaches.

*I think it does depend on the individual people*,* how stable they are or not*,* how much they are open to looking at something different*.

Participation in SRT was enhanced when there were a combination of client readiness and the perceived relevance of SRT approach.

*He was right into it*,* and he was just the model patient…he was trying to make some changes*,* and he could see the sense of it*,* it really fitted in with him*.

In contrast, there was also reluctance from clients despite active efforts made by CMs to engage them in the process.

*I think recruiting people was quite hard…we give people the consent form and then one person in particular*,* I tried her three times*,* but in the end*,* she didn’t come through.*

*Navigating dual roles* described the reality of integrating delivery of SRT into the CM role.

There was a clear tension between SRT therapy delivery and CM role reflecting role strain. One of the keys factors was their overall workload and their capacity to do all the things required of them describing *“the work just keeps piling up”.* The workload issues extended beyond the individual CM and reflected a broader systemic context as described by this CM:

*I just can’t be in two places at once. It’s just been really difficult because I think the team’s under so much stress.*

The level of work demands compromised the delivery of SRT affecting the frequency of contact with their clients and disrupting therapeutic momentum.

*When we got onto a session it was like where were we last and then sort of picking up from there to move forward again so it was very sort of stop/start*.

Whilst CMs felt SRT was beneficial and were in favour of it being implemented, there was a misalignment between delivering therapy and CM role.

*I think it was just the clash with therapy versus case management and if there was differentiation between the two… it would have been a lot easier than trying to case manage the chaos as well.*

Delivering SRT was deemed to require a different approach with the suggestion of dedicated specialist roles may be more effective.

*If you had someone just doing that for bipolar clients*,* just specialising doing that*,* I think you would get through.*

*Beyond medication* reflected the impact of having access to a psychological based intervention. There was clear reflection of the value of SRT as a non-medication-based intervention and an alternative to the predominant medication focus to treatment.

- *I think it was quite helpful from that framework*,* really and I think for him because it’s probably for many years been focused around his medications.*
- *I just think it’s a really good way to support people…because it’s not really all about medications*.

SRT was valued for fostering understanding with the strong focus on the circadian understanding of BD a key beneficial element in giving clients with a different approach to make sense of their experiences of BD.

*The best aspects that most people got were around the circadian rhythms*,* the structure and understanding for themselves the fragility that stress can cause*.

Additionally, the application of SRT techniques promotes a client’s sense of agency and self-efficacy as reflected here:

*Everything he just did*,* and then when COVID hit*,* he went back to the old patterns of staying up to 2 o’clock in the morning and not getting up to midday*,* he got really down… we went to the basics of what we did*,* the routine*,* about getting up*,* having goals…and he turned himself around in a matter of two or three weeks…for him*,* it was really good because he could actually see that this actually works…he said I think I know if something happens again*,* I know what to do.*

SRT was perceived as a potentially valuable therapeutic intervention to deliver and warranting continued implementation.

*It’s certainly something that I will continue to use… I think there are certainly some people who would benefit from doing the whole of SRT*.

However, this was tempered by the broader challenge of implementing within the reality of the work context.

*I think it would be really good in an ideal world. If all case managers could be doing it*,* I think it would be really good.*

### Treatment Adherence and Fidelity

Adherence to SRT was based on the case presentations at fortnightly supervision where the content and structure of model was monitored. The data tracking sheets confirmed numbers of sessions, length of session and duration for clients who completed therapy (*n* = 5) and those who discontinued therapy (*n* = 5) were consistent with manual protocol. Fidelity was unable to be assessed as only one CM completed the requirements for a taped session from beginning, middle and end of therapy.

### Discussion

The aim of this study was to examine the feasibility of implementing SRT for individuals with BD receiving outpatient care in an SMHS within a public health system. While this study demonstrated that it was feasible to train CMs in SRT with a combination of initial training and ongoing supervision, its implementation into routine practice was problematic despite the study being initiated and supported by SMHS personnel. This was primarily due to the demands of the CM role impacting on their ability to effectively deliver SRT compounded by broader resourcing within the service. Low recruitment number suggested acceptability to individuals with BD was limited; however, from the perspective of the CMs, those who received SRT found it beneficial.

Despite the evidence supporting the potential of SRT as a translatable therapy for individuals with BD (Crowe et al. 2020a), our findings reflect the challenges in the adoption of evidence-based treatments in complex systems such as mental health services. Organisational factors related to the CM role, workplace demands, and the dominant frameworks of care were significant barriers to implementation which have previously been found in implementing interventions in mental health inpatient settings (Raphael et al., 2021). Successful implementation requires higher level system buy-in including at resourcing decision making levels. Funding decisions need to consider the significant economic burden associated with BD including direct healthcare costs`, particularly inpatient care`, and indirect costs such as unemployment and caregiver productivity loss (Bessonova et al., 2020; Young et al., 2011) weighted up with the financial positive benefits of more effective treatments for those with BD (Miklowitz & Scott, 2009; Shields et al., 2019).

CMs compromise a substantive workforce within mental health services and enhancing their capacity to deliver evidence-based therapies as part of their role has demonstrated promise (Ravitz et al., 2019). Our findings reflect the challenges of this approach given the multiple demands placed on the CM role, which impeded their ability to successfully implement SRT. Previous concerns about the CM role have been identified, including being a poorly defined generic role (Lukersmith et al., 2016), subject to increased requirements with resultant increased burden, less clarity, and increased demands over time (Owen, 2014), and often focused on risk management and the promotion of medication adherence (Crowe & Carlyle, 2003) rather than providing therapeutic interventions. An alternative is the development of more specialised roles for some CM as has been achieved in the treatment of individuals with borderline personality disorder (Carlyle et al., 2020). However, our findings suggest that incorporating evidence-based interventions more clearly into their role would require a significant shift in the model of service delivery. One such approach is a system-based response, the Therapy Capability Framework, developed to enhance case management roles in the provision of evidence based psychological therapies within mental health services which focuses on identifying core capability domains in implementing psychological therapies, building competency, and active support from clinical and service leadership. (Lau et al., 2017).

Our finding of low uptake of individuals with BD reflected a range of factors impacting their decision and require more in-depth understanding. Similarly, Bradley et al. (Bradley et al., 2013) had challenges in their IPSRT implementation study. Despite treatment guidelines indicating improved outcomes for people with BD require a combination of medication and evidence-based psychological intervention (Malhi et al., 2021; Yatham et al., 2018), there remains a predominance of the biomedical approach, the centrality of pharmacological interventions, and a lack of exposure to psychological interventions. This combination may influence people’s willingness to engage and openness to differing frameworks. However, this contrasts with evidence from a meta-analysis identifying high patient preference for access to psychological interventions (Kathryn McHugh et al., 2013). Additionally, alternatives and adjuncts to pharmacology have been identified as a priority by individuals with BD (Nestsiarovich et al., 2017). A further factor potentially impacting on uptake may relate to the perception of the disciplines who provide therapy with psychologists clearly playing a key role in this (Hunt et al., 2025). However, limiting delivery to one discipline creates challenges in scaling access to treatment hence a need to diversity the workforce able to provide the interventions. (Kazdin, 2017).

An additional finding was the challenges of conducting research within clinical services, as evidenced by the lack of client data, and limited CM data. The issue of research burden has been previously highlighted with evidence suggesting this is a barrier to recruitment in research (Ulrich et al., 2005). A higher level of engagement of key stakeholders in the research design, in particular involving those with lived experience, and potential recipients of the intervention may improve participation and willingness to undertake research-related activities (Corrigan et al., 2001; Riches et al., 2023).

There were several limitations related to the feasibility nature of our study. These include a small sample size, only focused on two community teams within SMHS, and significant gaps in data, especially reflecting the clients’ perspectives. This clearly limits the generalisability of our findings. Despite this, it does highlight important factors to consider in future studies addressing the critical challenge of implementing evidence-based intervention within usual care settings.

Improving outcomes for people with BD requires a continued commitment to research that addresses implementation and systemic uptake of evidence-based interventions (Deenik et al., 2019). Enhancing their adoption in SMHS requires system change and openness at all levels to alternative approaches entailing involvement of a range of stakeholders at various levels in the organisation including people with BD in the design and implementation process. Based on our findings, the key areas would be to address the CM role and identify ways to enhance their capacity and capability to deliver evidence-based interventions, and secondly, to embed a framework that prioritises access to adjunctive psychological interventions.

### Conclusion

There are obvious gaps between clinical guidelines and the reality of everyday practice. Our findings reflected the challenges associated with implementing a brief, evidence-based intervention delivered by CMs into a SMHS. Factors associated with the models of care and resourcing of mental health services impeded the delivery of SRT. Addressing the issue at a system and structural level is needed to achieve increased access to evidence based therapies for individuals with BD in SMHS. 

## Data Availability

No datasets were generated or analysed during the current study.
